# Broad-Spectrum Antimicrobial Potential of the γ-Core Motif Peptides of *Filipendula ulmaria* for Practical Applications in Agriculture and Medicine

**DOI:** 10.3390/ijms26167959

**Published:** 2025-08-18

**Authors:** Marina P. Slezina, Ekaterina V. Kulakovskaya, Ekaterina A. Istomina, Tatiana N. Abashina, Tatyana I. Odintsova

**Affiliations:** 1 Vavilov Institute of General Genetics RAS, 119333 Moscow, Russia; omey@list.ru (M.P.S.); mer06@yandex.ru (E.A.I.); 2 Federal Research Center “Pushchino Scientific Center for Biological Research of the Russian Academy of Sciences”, G.K. Skryabin Institute of Biochemistry and Physiology of Microorganisms RAS, 142290 Pushchino, Russia; ekaterina.kulakovskaya@gmail.com (E.V.K.); tnabashina@gmail.com (T.N.A.)

**Keywords:** plant antimicrobial peptides, γ-core, novel antimicrobials, plant pathogens, human pathogens

## Abstract

Antimicrobial peptides (AMPs) are the promising candidates for the development of next-generation antimicrobials for agriculture and medicine; however, their large-scale production is costly. The γ-core motif peptides, functionally significant fragments of AMPs responsible for the antimicrobial activity, provide a more economical and feasible approach for the commercial development of novel antimicrobials. In the present work, we undertook a comprehensive study of antimicrobial properties of several γ-core peptides derived from defensins and snakins of *Filipendula ulmaria*, a medicinal plant known for its valuable pharmacological properties. The γ-core peptides were produced by solid-phase synthesis and purified by RP-HPLC. Their physicochemical properties underlying biological activity were predicted. All the peptides ranging in size from 14 to 18 amino acid residues were positively charged. All peptides except one were predicted to be α-helical and antimicrobial. The synthetic peptides were in vitro tested against a wide panel of plant and human fungal and bacterial pathogens. A short overview of the pathogens used in antimicrobial assays with a special emphasis on their economic, social, and medicinal impacts is provided. As a result of our work, we identified the peptides with pronounced activity in low-micromolar range against particular pathogens that can serve as prototypes for the development of novel biopesticides and antimicrobials for medicine. We also revealed synergism of action between particular γ-core peptide pairs and demonstrated that interference with membrane permeabilization contributes to the peptides’ mode of action. The results obtained broaden our understanding of plant AMPs, the key players in plant immunity, and provide novel highly efficient peptides with high potential in practical applications.

## 1. Introduction

Plant diseases provoked by pathogenic microorganisms (fungi, oomycetes, bacteria, and viruses) pose a significant threat to crop production worldwide. They cause dramatic pre- and postharvest yield losses and deteriorate the quality of food, which is of paramount concern especially in low-income countries. Persistent yield losses due to plant diseases can reach 20% of the world’s harvest, with a further 20–25% postharvest loss [1,2].

Fungi and oomycetes are among the dominant causal agents of plant diseases [3,4]. Fungal plant pathogens destroy crop products, while mycotoxin-producing and food-spoiling fungi further decrease the supply of safe foods [5,6]. Fungi are a major cause of postharvest deterioration of cereals, legumes, fruits, and oil seeds [7]. Crop loss caused by molds during storage can be very high, the estimates showing that about one third of all produced food is lost due to molds [8]. Besides plants, fungi can infect humans and cause various diseases, from skin infections to invasive infections of internal organs [9]. Invasive infections lead to life-threatening diseases, especially in immunocompromised patients, and kill one and a half million people every year [10]. Several hundred fungal species are associated with human diseases, among them, *Candida albicans*, *Cryptococcus neoformans*, *Aspergillus fumigatus*, *Histoplasma capsulatum*, etc. [9]. Of interest, some plant pathogenic fungi can cause diseases in humans as well. A remarkable example is the *Fusarium* species capable of infecting plants, animals, and humans, a phenomenon known as trans-kingdom pathogenicity [11,12].

While about 10,000 species of fungi and oomycetes cause diseases in plants, the estimated number of plant pathogenic bacteria is about 200 species [13,14]. However, most important agricultural crops suffer from at least one bacterial disease, and for some crops, bacterial disease is the main cause of yield losses. Consistent annual crop losses are recorded in all countries, although bacterial diseases are most severe in tropical countries, where warm and humid climate favors rapid bacterial growth [15]. The most deleterious bacterial species belong to the genera *Xanthomonas*, *Pseudomonas*, and *Erwinia* [16].

With population growth, climate change, and reduced land resources, the need for high-quality food will increase, which reinforces the need for effective disease management. The use of pesticides remains the prevalent strategy of disease control [17]. Pesticides play a crucial role in enhancing crop yields and food quality. However, the use of pesticides has negative effects on the environment and human health. Pesticides can be toxic to non-target organisms, such as birds, fish, beneficial insects, and plants. They pollute soil, water, and air. In addition, the production and application of fungicides contribute to CO_2_ emissions. Among the problems of prime importance are harmful effects on human health. Pesticide exposure induces chronic diseases including cancer, neurodegenerative diseases, birth defects, and asthma [18]. Last but not the least, the use of pesticides induces resistance emergence in pathogens, which requires continuous development of new pesticides with a different mode of action. All this calls for the search and development of new environmentally safe strategies of plant protection against diseases.

In contrast to chemical pesticides, biopesticides offer several advantages over conventional chemical pesticides: they show low or no toxicity to non-target organisms; they have low environmental impacts; their biodegradability reduces pollution; in addition, there is a lower likelihood of developing resistance in pathogens [19,20]. Plant-derived biopesticides already used in crop protection are plant extracts and essential oils [8,21,22,23]. Several products for crop protection based on plant extracts are already on the market: Vertigo^®^ (Monterey Laboratories, Watsonville, CA, USA) made from the seeds of *Cassia obtusifolia*, Milsana^®^ (Biofa GmbH, Münsingen, Germany) from *Reynoutria sachlinesis*, and Qwel^®^ (Camas Technologies Ltd., Rovato, Italy) prepared from the extract of *Macleaya cordata* [24].

Antimicrobial properties of essential oils and plant extracts have been mainly attributed to phenolic compounds [25]. The polypeptide-based molecules present in plants are much less studied. Antimicrobial peptides (AMPs) represent a valuable and incompletely explored source of diverse molecules with antimicrobial properties with a high potential for application in agriculture as innovative biopesticides and in medicine as next-generation antibiotics.

AMPs comprise an evolutionary ancient mechanism to combat pathogens, which proved highly effective in the arms race between plants and pathogenic microorganisms. AMPs are small polypeptides consisting of up to 100 amino acid residues enriched in charged and hydrophobic residues that form distinct patches on the AMP molecule [26]. This peculiarity underlies the ability of AMPs to interact with negatively charged membranes and the cell walls of the pathogenic microorganisms, mitigating their integrity and, in some instances, providing entry to intracellular targets. Disruption of the cell walls and membranes of the pathogens is a main antimicrobial mechanism of AMPs [27]. Interaction with diverse molecules inside the cells is another mechanism of pathogen suppression. AMPs display a variety of activities, from antimicrobial to immunomodulatory, and show modes of action distinct from those of single-site antibiotics and fungicides. Comparative studies revealed obvious advantages of AMPs over conventional antibiotics and fungicides: they act quicker, they are less prone to resistance development, and their natural origin and rapid degradation reduce environmental persistence compared to synthetic pesticides [28].

Every plant species harbors hundreds of AMP-like genes [29,30]. In response to pathogen attacks, they are differentially expressed, providing a finely tuned immune reaction to the invasion. The arrays of AMPs present in each plant form an invaluable resource of biologically active molecules for plant protection and human disease control. Medicinal plants with antimicrobial and anti-inflammatory properties are of particular interest. *Filipendula ulmaria*, a perennial herb of the Northern hemisphere, has been widely used in folk medicine to treat various diseases, such as colds, conjunctivitis, arthritis, and rheumatism. Meadowsweet is renowned for its wide-ranging pharmacological activities, including antioxidant [31,32,33,34], gastroprotective [35], and anti-cancer effects [34,36,37,38]. It also exhibits neuroprotective properties [31,39,40], alongside enhancing skin barrier function and promoting its renewal [41]. The antimicrobial properties of meadowsweet have been convincingly demonstrated, making this medicinal plant a promising candidate for developing natural antimicrobials [33,42]. The plant’s diverse phytochemicals—secondary metabolites—were shown to contribute to these activities, while polypeptide-based molecules, AMPs in particular, and their role in the biological activities of meadowsweet remain unexplored. In our previous work, to retrieve candidate molecules with antimicrobial properties, we explored the differential expression of AMP-like genes in meadowsweet in response to infection with *Bipolaris sorokiniana*, a causal agent of devasting diseases of cereals, and identified the up- and down-regulated genes [43]. The former were likely to present novel antimicrobials.

In the present work, we undertook a comprehensive study of antimicrobial properties of several previously identified *F. ulmaria* AMPs. For this purpose, we synthesized the peptides corresponding to their γ-core regions implicated in antimicrobial activity and tested the synthetic peptides against a wide panel of plant and human fungal and bacterial pathogens. As a result, we identified the peptides with pronounced activity against particular pathogens, which can serve as prototypes for the development of novel biopesticides and antimicrobials for medicine.

## 2. Results

### 2.1. Design of γ-Core Peptides

**Figure 1:**
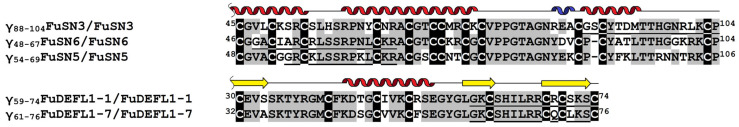
Multiple sequence alignment of the γ-core-containing regions of meadowsweet AMPs. Superscript numbers show their location in the precursor proteins. The sequences of synthetic peptides are underlined. The designations of synthetic peptides and their parent AMPs are provided on the left. Cysteine residues are black, and identical amino acids are gray. Secondary structure elements (α- and 3_10_-helices, and β-strands) are marked above the corresponding sequences as helices (α-helix is shown in red, and 3_10_-helix is colored blue) and arrows, respectively.

Meadowsweet AMPs discovered in the transcriptomes of plants infected by *B. sorokiniana* were analyzed for the presence of γ-core sequences [43]. The γ-core motifs GXCXnC were found in defensin-like peptides (DEFLs), snakins, non-specific lipid-transfer proteins, and MEG peptides. Our previous work showed that peptides homologous to the γ-core sequences of defensins and snakins had the highest activity against pathogens [44,45]. It should be specifically noted that snakin γ-core sequences lack the characteristic 3D structure of classical γ-cores, which consists of two antiparallel β-strands connected by a short turn (Figure 1). Among the γ-core-containing DEFLs and snakins, two DEFLs and three snakins of *F. ulmaria* were chosen for the synthesis of the γ-core motif peptides (Figure 1).

Two γ-core motif peptides, γ_59-74_FuDEFL1-1 and γ_61-76_FuDEFL1-7, were designed from the sequences of classical defensins, FuDEFL1-1 and FuDEFL1-7, respectively. The synthetic γ-core peptides of the classical defensins in addition to the γ-core motif contained a C-terminal region, including two additional cysteine residues (Figure 1). The peptides showed high sequence similarity and differed by two residues, Q11R and L13S. The FuD0 peptide, a hybrid variant of the homologous γ_59-74_FuDEFL1-1/γ_61-76_FuDEFL1-7 peptides and the previously studied highly active wheat peptide DEFL1-16_65-82_ with the amino acid sequence GKCRGFRRRRCFCTTHCHH [44], was also selected for synthesis. FuD0, like DEFL1-16_65-82_, had Phe, Thr, Thr, and His at positions 11, 13, 14, and 15, respectively (Table 1).

Of the seven snakins discovered in *F. ulmaria* transcriptomes, only three peptides possessed a γ-core motif. FuSN3 contained the γ-core motif at the C-terminal region, while FuSN5 and FuSN6 contained it at the N-terminal regions of the molecules (Figure 1). The synthetic γ-core peptide of FuSN6, named γ_48-67_FuSN6, in addition to the γ-core motif GXCXnC contained one additional cysteine residue and a C-terminal region homologous to the FuSN5 γ-core peptide—γ_54-69_FuSN5 (Figure 1).

The γ-core-containing peptide γ_89-106_SlSN9 derived from the tomato snakin SlSN9 and described previously [45] was selected for comparison (Table 1).

Thus, five short peptides encompassing the γ-core regions of AMP-like peptides of meadowsweet and one modified analogue with substitutions at the C-terminus, as well as the _γ89-106_SlSN9 peptide, were obtained by solid-phase synthesis and purified by high-performance liquid chromatography (Table 1).

### 2.2. Physicochemical Properties of Meadowsweet Synthetic Peptides

The calculated physicochemical properties of *F. ulmaria* synthetic peptides are presented in Table 2. The peptides contain from 14 to 18 amino acid residues. Their molecular weights are in the range from 1632.02 to 2004.42 Da. All of them are cationic. The pI (isoelectric point) values vary from 8.05 in γ_88-104_FuSN3 to 11.02 in γ_54-69_FuSN5. The net charge is in the range from +1 to +6 (Table 2). The highest positive charge of +6 in γ_54-69_FuSN5 is due to the presence of six positively charged residues (Arg or Lys). In γ_59-74_FuDEFL1-1 and γ_48-67_FuSN6, there are five positively charged residues, resulting in the net charge of +5. γ_61-76_FuDEFL1-7 and FuD0 contain four and three positively charged residues, resulting in the net charge of +4 and +3, respectively. In γ_88-104_FuSN3, there are two positively charged residues and a single negatively charged residue, resulting in the lowest net charge of +1. The positive charge of the molecule is necessary for the electrostatic interaction of the peptide with the negatively charged cell membranes [46]. The aliphatic index, which displays a positive correlation with thermal stability, is in the range from 22.94 to 76.11, being the highest in γ_48-67_FuSN6 (Table 2). The Boman index reflecting protein-binding potential varies from 2.14 in FuD0 to 3.91 in γ_54-69_FuSN5. Half of the γ-core peptides have Boman index values above 2.5; these values indicate that these AMPs can interact with a wide range of proteins. The ratio of hydrophobic amino acid residues ranges from 24% in γ_88-104_FuSN3 to 44% in γ_48-67_FuSN6, γ_61-76_FuDEFL1-7, and FuD0. Hydrophobicity, which exceeds the average portion of hydrophobic residues in AMPs of 41.5%, promotes the insertion of the peptide into the microbial membrane lipids. The GRAVY (Grand Average of Hydropathy) index is negative for all peptides (Table 2). Negative values of the GRAVY index characterize peptides that are more hydrophilic and dissolve better in water. All peptides except γ_88-104_FuSN3 were predicted to be antimicrobial.

### 2.3. Three-Dimensional Structure Modeling of Synthetic Peptides

**Figure 2:**
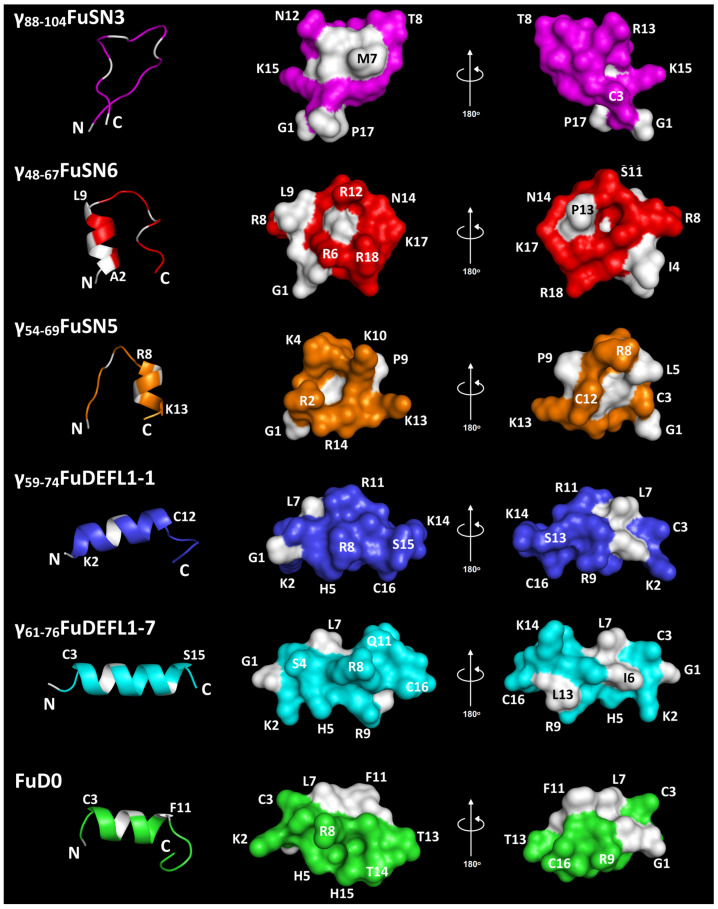
Three-dimensional molecular modeling of the meadowsweet synthetic peptides: spatial (ribbon representation) and surface structures. N- and C-termini are marked with N and C, respectively. Non-polar residues are shown in white, and polar residues are colored. Modeling was performed using PEP-FOLD3 [47].

The 3D structure of synthetic peptides was predicted with PEP-FOLD 3 (Figure 2) [47]. γ_88-104_FuSN3 was predicted to be in a random coil conformation, while other five peptides contained an α-helical region of different length (from 7 to 13 amino acid residues) and unstructured N- and C-terminal «tails». In γ_59-74_FuDEFL1-1 and γ_61-76_FuDEFL1-7, the α-helical region was predicted to occupy most of the peptide’s molecule. The longest α-helix of 13 residues was predicted in γ_61-76_FuDEFL1-7. In FuD0, the helical region is located in the middle of the peptide. γ_61-76_FuDEFL1-7 and γ_54-69_FuSN5 have a short α-helix. In γ_61-76_FuDEFL1-7, it is in the N-terminal region of the molecule followed by an unstructured C-terminal region, while γ_54-69_FuSN5 contains it in the C-terminal region. Most peptides are amphipathic with hydrophobic and polar residues located on the opposite sides of the molecule. Moreover, positively charged residues are grouped together forming a distinct cluster: R6, R12, K17, and R18 in γ_48-67_FuSN6; R2, K4, R8, K10, K13, and R14 in γ_54-69_FuSN5; K2, H5, R8, and R9 in γ_61-76_FuDEFL1-7; K2, H5, R8, R9, and H15 in FuD0; and H5, R8, R9, and R11 in γ_59-74_FuDEFL1-1.

### 2.4. Antimicrobial Activity of Synthetic Peptides Against Yeasts and Bacteria

The antimicrobial activity of synthetic peptides was screened against a panel of pathogens causing diseases in plants and humans: the bacteria *Clavibacter michiganensis*, *Pseudomonas savastanoi*, and *Pectobacterium carotovorum* and the yeasts *Candida albicans*, *C. tropicalis*, and *Cryptococcus neoformans*.

Studies of the antimicrobial activity of seven synthetic peptides showed that all of them suppressed the growth of yeasts *C. neoformans* and *C. tropicalis*; however, the efficiency of inhibition was different depending on the peptide tested (Table 3). γ_54-69_FuSN5 and γ_61-76_FuDEFL1-7 were the most potent against *C. neoformans* (IC_50_ = 1.4 and 2.7 μM, respectively). The peptides γ_48-67_FuSN6, FuD0, and γ_89-106_SlSN9 were slightly less active, with IC_50_ values of 5.5, 6.1, and 6.3 μM, respectively. γ_48-67_FuSN6, γ_54-69_FuSN5, and γ_61-76_FuDEFL1-7 showed high antimicrobial activity against *C. tropicalis* (IC_50_ = 1.7, 2.5, and 3.6 μM, respectively). FuD0, γ_89-106_SlSN9, and γ_59-74_FuDEFL1-1 inhibited *C. tropicalis* with lower efficiency (Table 3). Of the pathogenic yeasts, *C. albicans* was the least sensitive to the AMP-derived peptides. γ_54-69_FuSN5 and γ_59-74_FuDEFL1-1 peptides showed the best results in inhibiting this pathogen (IC_50_ = 10.4 and 13.8 μM, respectively).

The AMP-derived peptides were able to suppress the growth of two bacteria, Gram-positive *C. michiganensis* and Gram-negative *P. savastanoi*, with varying efficiency and failed to suppress the growth of *P. carotovorum* at the tested concentrations. γ_48-67_FuSN6 and γ_89-106_SlSN9 showed the best activity against *C. michiganensis* (IC_50_ = 34.7 and 41.9 μM, respectively). The FuD0 of all the peptides was the most potent against *P. savastanoi.* All other peptides showed much lower inhibitory activity, while γ_88-104_FuSN3 failed to inhibit the growth of all tested bacterial species. It should be noted that γ_88-104_FuSN3 activity against yeasts was also weak. This is probably related to its low net charge (Table 2).

### 2.5. Antimicrobial Activity Against Filamentous Fungi

In contrast to yeasts, the activity of AMP-derived peptides against filamentous fungi (*Aspergillus unguis*, *Bipolaris sorokiniana*, *Botrytis cinerea*, *Rhizoctonia solani*, *Penicillium gladioli*, *Fusarium oxysporum*, *F. culmorum*, *F. solani*, and *F. verticillioides*) was lower (Table 4). Of the *Fusarium* species, *F. oxysporum* was inhibited to the greatest extent; the IC_50_ values of FuD0 and γ_48-67_FuSN6 against this pathogen were 11.3 and 15.8 μM, respectively. γ_59-74_FuDEFL1-1 was slightly less active, with IC_50_ of 22.2 μM. The peptides γ_48-67_FuSN6, γ_59-74_FuDEFL1-1, and γ_89-106_SlSN9 showed similar activity against *F. culmorum*; their IC_50_ were 19.3, 19.4, and 23.1 μM, respectively; and, in general, *F. culmorum* was inhibited by all the peptides quite efficiently (Table 4). *F. solani* was most strongly inhibited by FuD0 (IC_50_ = 17.7 μM). Against *F. verticillioides*, the γ_89-106_SlSN9, γ_59-74_FuDEFL1-1, and γ_48-67_FuSN6 peptides showed the highest activity (IC_50_ = 32.3, 36.1, and 38.0 μM, respectively). Of all the peptides, γ_88-104_FuSN3 had the lowest inhibitory activity against *Fusarium* fungi; at the concentrations tested, it showed very low activity against *F. verticillioides* and was inactive against other fungi. Among the other five tested fungal species, *A. unguis* was the most sensitive. The IC_50_ values of the peptides ranged from 2.1 μM for γ_48-67_FuSN6 to 37.0 μM for γ_61-76_FuDEFL1-7. γ_88-104_FuSN3 and γ_89-106_SlSN9 also possessed high inhibitory activity (IC_50_ = 2.2 and 3.3 μM, respectively). Against *B. sorokiniana*, γ_59-74_FuDEFL1-1 and γ_48-67_FuSN6 were the most active, with IC_50_ values of 45.0 and 46.2 μM, respectively. The activity of the peptides against *P. gladioli* was relatively low, with the IC_50_ values exceeding 67.3 µM. *R. solani* and *B. cinerea* were the most resistant to the action of the peptides (Table 4). The best activity was displayed by γ_88-104_FuSN3; its IC_50_ against *B. cinerea* was 61.7 µM.

### 2.6. Synergistic Interactions of the AMP-Derived Peptides

Two pairs of peptides were selected to reveal possible synergism of action: γ_59-74_FuDEFL1-1/γ_61-76_FuDEFL1-7—a pair of defensin-derived peptides; and FuD0/γ_54-69_FuSN5—a pair of defensin- and snakin-derived peptides. Synergism of action was tested on two different pathogens: the fungal pathogen *F. oxysporum* and the bacterium *C. michiganensis*.

The activity of the peptides of the first pair against *F. oxysporum* was approximately the same, with γ_59-74_FuDEFL1-1 being slightly more active than γ_61-76_FuDEFL1-7 (Table 4 and Table 5). In almost all instances, the observed levels of inhibition in co-applications (Er) significantly exceeded the effects of the peptides alone (Table 5). To prove that the enhanced antifungal effect was associated with a synergistic interaction between the two peptides, and to identify combinations of concentrations of each peptide leading to synergism, the inhibitory effects observed when the peptides were used together were compared with the calculated additive effects of the peptides (Ee) (Table 5). As a result, it was shown that, in all cases, when the concentration of one peptide of the pair was less than 25 μM except one (10 µM γ_59-74_FuDEFL1-1 and 75 µM γ_61-76_FuDEFL1-7), Er values exceeded Ee values (Table 5). The same situation was observed for the combinations of 25 or 50 µM γ_59-74_FuDEFL1-1 and 25 µM γ_61-76_FuDEFL1-7. These differences were significant at *p* ≤ 0.05 (*t*-test for independent variables) for the following combinations of peptide concentrations: 25 µM γ_59-74_FuDEFL1-1 and 5 µM γ_61-76_FuDEFL1-7; and 5 or 10 µM γ_59-74_FuDEFL1-1 and 25 µM γ_61-76_FuDEFL1-7 (Table 5), which means that, at these combinations of peptide concentrations, the enhancement of antifungal activity was due to the synergistic action of peptides. In other cases, the peptides acted additively.

The peptides of the second pair had a larger gap in activity against *F. oxysporum*. The growth inhibition caused by γ_54-69_FuSN5 was only 54.7% at the peptide concentration of 150 µM, while FuD0 suppressed the growth of *F. oxysporum* by 64.9% at the concentration of 25 µM (Table 6). The co-application of these peptides also resulted in an enhanced antifungal effect compared to the action of the peptides taken alone, but Er values exceeded the calculated Ee values in fewer cases than for the γ_59-74_FuDEFL1-1/γ_61-76_FuDEFL1-7 pair: 5 µM FuD0 and 50, 75, 100, 150 µM γ_54-69_FuSN5; 10 µM FuD0 and 50, 75, 150 µM γ_54-69_FuSN5; 25 µM DuD0 and 5, 10, 25, or 75 µM γ_54-69_FuSN5; and 50 µM FuD0 and 25 or 75 µM γ_54-69_FuSN5 (Table 6). Moreover, only the combination of 5 µM FuD0 and 75 µM γ_54-69_FuSN5 resulted in a synergistic interaction at *p* ≤ 0.05; other combinations resulted in an additive effect.

The results of synergism evaluation for the two pairs of peptides studied against bacterium *C. michiganensis* are shown in Table 7 and Table 8. All the peptides had low inhibitory activity when tested individually. In many instances, the observed levels of inhibition in co-applications were higher than the effects of the individual peptides alone (Table 7 and Table 8). However, in contrast to *F. oxysporum*, the observed inhibitory effect against *C. michiganensis* did not exceed the calculated additive effect at any combination of peptide concentrations in the pairs γ_59-74_FuDEFL1-1/γ_61-76_FuDEFL1-7 and FuD0/γ_54-69_FuSN5, and thus, no synergism of action against *C. michiganensis* was detected.

### 2.7. Staining with Propidium Iodide

To study the mechanism of action, five meadowsweet synthetic peptides were used: FuD0, γ_59-74_FuDEFL1-1, γ_61-76_FuDEFL1-7, γ_54-69_FuSN5, and γ_48-67_FuSN6. Their ability to disrupt the integrity of the cytoplasmic membrane of pathogens was evaluated by determining the proportion of cells stained with propidium iodide (PI) after treatment with the peptides. The following pathogens were used for the examination: *C. albicans*, *C. tropicalis*, *C. neoformans*, and *C. michiganensis*. The results were quantified by flow cytometry.

**Figure 3:**
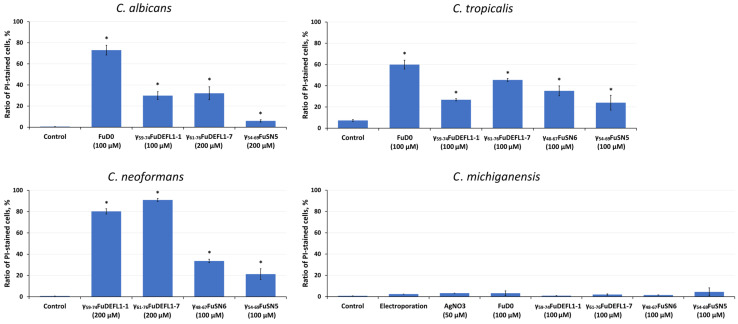
Penetration of propidium iodide (PI) into pathogen cells treated with the γ-core motif peptides. Untreated cells were used as the negative control. Bars show mean ± SD. Asterisks demonstrate significant differences between treated and control cells (Student’s *t*-test, *p* < 0.05).

All tested peptides triggered accumulation of the fluorescent dye inside the cells of three yeast species, which indicated permeabilization of the membranes as one of the mechanisms of the peptides’ action (Figure 3). *C. michiganensis* turned out to be an unsuitable object for such experiments, since these bacteria stained weakly with PI even after electroporation or treatment with 50 μM AgNO_3_ (Figure 3).

## 3. Discussion

Plant diseases caused by pathogenic microorganisms significantly impact agricultural productivity. They lead to substantial yield losses and diminished food quality, posing a major challenge to global food security. Chemical pesticides are widely employed in modern agriculture to manage pathogenic microbes, with global pesticide production experiencing an annual growth rate of approximately 11%, rising from 0.2 million tons in the 1950s to over 5 million tons by 2000 [48]. This trend reflects increased reliance on chemical control methods to protect crops and ensure food security. Growing awareness of the hazards posed by chemical pesticides to human health, animals, and the environment has spurred the search for environmentally friendly alternatives, notably plant-derived biopesticides. Plants, especially medicinal species, offer a rich reservoir of bioactive compounds with antimicrobial properties [49,50,51,52,53,54,55].

Antimicrobial peptides, natural defense molecules, are promising candidates for developing novel fungicides and antibiotics due to their broad-spectrum activity, rapid pathogen eradication, synergistic effects, lower resistance development, and minimal environmental and health impacts [28]. However, their large-scale production remains a challenge. Extraction from plant tissues yields limited quantities, while recombinant expression in heterologous hosts, like bacteria, yeast, insect cells, and plants, offers scalable solutions. Chemical synthesis, particularly solid-phase peptide synthesis, is also crucial, especially for modified peptides, but it is costly. To address this problem, researchers synthesize shortened, functionally essential fragments of AMPs that retain activity, providing a more economical and feasible approach for commercial biopesticide development.

Structure–function studies have identified the γ-core motif in AMPs as a crucial structural element largely responsible for antimicrobial activity [56]. It is characterized by a GXCX_3-9_C sequence forming a three-dimensional structure with two anti-parallel β-strands connected by a hairpin loop. The γ-core is both positively charged and amphiphilic, which promotes its interactions with membranes. It was shown that the mechanism of antimicrobial activity of γ-core peptides, in addition to the destruction of the plasma membrane, also includes inhibition of plasma membrane H^+^-ATPases in pathogens, thereby disrupting vital cellular processes [57]. Synthetic peptides mimicking this motif have demonstrated potent antimicrobial activity (reviewed in [58]). This discovery highlights the γ-core motif’s potential as a target for designing novel biopesticides and therapeutics, leveraging its conserved presence in many antimicrobial peptides and its key role in antimicrobial efficacy.

Based on our previous RNA-seq analyses that identified AMP-like genes in *F. ulmaria* [43], some of which were upregulated upon *B. sorokiniana* infection, this study extensively evaluates the antimicrobial activity of six γ-core motif peptides derived from *F. ulmaria* AMPs against various plant and human opportunistic pathogens, aiming to identify promising peptide candidates for developing innovative biopesticides and antimicrobial agents.

We synthesized six short peptides containing the γ-cores of *F. ulmaria* AMPs, and one peptide, γ_89-106_SlSN9, was included for comparison (Table 1). All meadowsweet γ-core peptides were derived from defensins and snakins. The synthetic peptides differed in their physicochemical properties (Table 2). All of them were positively charged. Positive charge facilitates interactions with the membranes of pathogens. γ_54-69_FuSN5 had the highest positive charge of +6, followed by γ_48-67_FuSN6 and γ_59-74_FuDEFL1-1 (+5) at a neutral pH. γ_88-104_FuSN3 had the lowest charge of +1. All peptides except γ_88-104_FuSN3 were predicted to adopt α-helical conformation with unstructured «tails» (Figure 2). γ_88-104_FuSN3 was in a random coil conformation.

The synthetic peptides were assayed against a panel of plant and opportunistic human pathogens. Among human pathogens, the inhibition of yeasts *C. albicans*, *C. tropicalis*, and *C. neoformans* by the peptides was analyzed. *C. neoformans* and *C. albicans* fall into the critical priority group according to the WHO classification of fungal pathogens based on several criteria, including incidence, antifungal resistance, mortality rates, and treatment difficulties, which aims to guide research and public health efforts on priority fungal pathogens [59].

*Candida* species (Ascomycota), with over 160 species identified in nature, are opportunistic pathogenic yeasts. Biofilm formation and associated virulence traits, such as adhesion, extracellular enzyme production, and hemolytic activity, are crucial for the pathogenicity of *Candida* spp., contributing significantly to their persistence in host tissues and resistance to treatment [60,61].

*C. albicans* causes a wide range of infections, particularly in immunocompromised individuals. Being part of the normal human microbiota, the fungus resides predominantly in the oral cavity, gastrointestinal tract, and vaginal mucosa. However, under certain conditions, such as immune suppression, antibiotic use, or disruption of mucosal barriers, *C. albicans* can overgrow and cause diseases known as candidiasis [62]. The infections range from superficial, localized conditions to invasive, systemic diseases such as candidemia, which can affect vital organs—the heart, brain, and kidneys. The global incidence of candidemia and invasive candidiasis has been increasing in the last decade, with estimates suggesting 250,000 to 700,000 cases annually. *C. albicans* remains the predominant species, responsible for approximately 40–50% of cases worldwide [63].

*C. tropicalis* has emerged as a significant opportunistic fungal pathogen, responsible for both superficial and severe invasive infections, ranking as one of the leading causes of candidemia in various patient groups [64]. *C. tropicalis* has also gained increasing attention, particularly in Asia-Pacific and Latin America, due to its widespread ecological presence and pathogenic potential, especially in cardiovascular infections like infective endocarditis. In contrast to *C. albicans*, which mainly associates with mammalian hosts, *C. tropicalis* is highly adaptable, thriving in diverse environments such as soil, sand, fruits, and plants, due to its remarkable ecological plasticity. *C. tropicalis* is notable for its ability to resist antifungal drugs, especially azoles, utilizing the mechanisms like efflux pumps and altered drug metabolism.

*C. neoformans* (Basidiomycota) is an opportunistic pathogenic yeast responsible for over 180,000 annual deaths worldwide, primarily affecting severely immunocompromised individuals [65]. The fungus is widespread in nature and is commonly found in soils contaminated with bird droppings and decaying plant materials. *C. neoformans* initially infects the lungs and then the fungus can disseminate to other organs and cause severe, life-threatening conditions like meningitis. Cryptococcal meningitis is a severe, often fatal disease if not diagnosed and treated promptly. The current treatment strategies for cryptococcosis are often inadequate, ineffective, or unavailable, particularly in developing countries. This highlights the critical need to develop more effective, accessible therapies to combat this life-threatening infection.

Studies of the antimicrobial activity of the synthetic peptides against yeasts revealed that the snakin-derived peptides were the most active (Table 3). γ_54-69_FuSN5 possessed the highest activity against *C. neoformans* (IC_50_ = 1.4 μM) and *C. albicans* (IC_50_ = 10.4 μM), while γ_48-67_FuSN6 was the most efficient in inhibiting *C. tropicalis* (IC_50_ = 1.6 μM). The defensin-derived peptide γ_61-76_FuDEFL1-7 was also highly efficient against *C. neoformans* (IC_50_ = 2.7 μM) and *C. tropicalis* (IC_50_ = 3.6 μM). Thus, the snakin-derived γ-cores γ_54-69_FuSN5 and γ_48-67_FuSN6, with the highest charge of the molecules, displayed the highest potency in yeast inhibition. γ_61-76_FuDEFL1-7 was more active than γ_59-74_FuDEFL1-1, although its net charge was lower; however, it was more hydrophobic (Table 2). Thus, both the charge and hydrophobicity contribute to the antimicrobial activity of the peptides against yeasts. γ_88-104_FuSN3 with the lowest charge and hydrophobicity was the least active peptide.

The γ-core peptides were also tested against two mold species—*A. unguis* and *P. gladioli*.

*A. unguis* (Ascomycota) inhabits soils and marine organisms, like jellyfishes and sponges. Although reports of *A. unguis* infections are rare and its pathogenicity remains uncertain, its frequent presence in households of severely asthmatic children suggests potential health risks, especially if inhaled in large concentrations, as the fungus can contribute to respiratory conditions like asthma or pneumonitis [66]. The primary concern with *Aspergillus* species generally stems from their ability to produce mycotoxins such as gliotoxin, aflatoxin, and ochratoxin A, which are associated with serious health problems, although there is currently no direct evidence that *A. unguis* produces these toxins [67]. In Finland, *A. unguis* was found to colonize water-damaged construction materials and produce sterigmatocystin, a potent carcinogenic and mutagenic mycotoxin, highlighting potential health risks associated with mold contamination in damp environments [68]. Diseases of the nails and skin associated with *A. unguis* have been reported [69,70]. Recent research has identified the *Aspergillus* species as emerging causative agents of non-dermatophyte mold onychomycosis. These emerging species involve *A. tubingensis*, *A. niger*, and *A. unguis*, which are notable for their ability to invade nail tissues and often pose diagnostic and therapeutic challenges due to their resistance profiles and the difficulty in distinguishing them from other molds.

The genus *Penicillium* (Ascomycota) encompasses multicellular fungi known for ecological plasticity and resistance to harsh environments, with many species utilized in food and pharmaceutical industries. However, some species are pathogenic, causing mold and rot in various plants. *P. gladioli* infects gladiolus and lily bulbs. *P. gladioli* also produces patulin, a polyketide mycotoxin [71]. It was initially used as an antibiotic against Gram-positive and Gram-negative bacteria, but is now recognized as toxic due to its affinity for sulfhydryl groups, inhibiting essential enzymes.

The growth of *A. unguis* was most effectively inhibited by the snakin-derived peptides γ_48-67_FuSN6 and γ_88-104_FuSN3 and the γ-core of tomato snakin γ_89-106_SISN9, with IC_50_ values as low as 2.1, 2.2, and 3.3 μM, respectively (Table 4). *P. gladioli* was less sensitive to the action of peptides. It was most efficiently suppressed by γ_59-74_FuDEFL1-1 and γ_88-104_FuSN3 (IC_50_ = 67.3 and 69.7 μM, respectively).

In this study, the synthetic γ-core peptides were tested against three plant pathogenic bacterial species: *C. michiganensis*, *P. savastanoi*, and *P. carotovorum.*

*C. michiganensis*, a Gram-positive actinobacterium, is a specialized plant pathogen with a very narrow host range, primarily infecting tomato in both field and greenhouse settings [72,73]; however, recent research indicates that subtle genomic variations among strains influence host specificity, and *C. michiganensis* has been sporadically reported to infect other solanaceous plants such as pepper (*Capsicum annuum*), eggplant (*Solanum melongena*), and wild nightshade species including *Solanum douglasii*, *S. nigrum*, and *S. triflorum* [74]. *C. michiganensis* causes bacterial canker of tomato, a globally widespread disease, which is considered one of the most significant bacterial threats to tomato cultivation together with bacterial spot and bacterial wilt [74]. The disease can cause severe yield losses, with plant mortality amounting to 93% and reductions in fruit weight averaging around 50%. The pathogen systemically infects tomato xylem causing symptoms like unilateral leaflet wilt, leaf necrosis, stem and petiole cankers, and ultimately plant death [75]. The bacterium also disperses to fruit surfaces, producing characteristic bird’s-eye lesions with necrotic centers and white halos, and can infect developing seeds through xylem colonization or by penetrating fruit tissues externally. Currently, there are no resistant tomato cultivars available, and control options like bactericidal sprays are limited in effectiveness since the pathogen invades the plant’s vascular system.

*P. savastanoi* pv. *savastanoi*, a Gram-negative bacterium, causes olive knot disease, which is a major threat to olive cultivation worldwide, leading to tumorous galls on the stems and branches of the host plant and, occasionally, on leaves and fruits that impair tree health and reduce yield [76]. Knots of infected trees host a complex bacterial microbiome that exacerbates disease severity [77]. Since eradication is nearly impossible once established, management relies heavily on preventive measures, including sanitary practices and cultural controls, complemented by regular copper applications over multiple years to suppress bacterial populations. Although some olive cultivars show varying susceptibility, no fully resistant varieties are currently available, emphasizing the importance of integrated disease management strategies to mitigate economic losses in olive groves.

Another Gram-negative bacterial pathogen, *Pectobacterium carotovorum* subsp. *carotovorum* (*Erwinia carotovora* subsp. *carotovora*), causes soft-rot disease and mainly affects crops in subtropical and temperate regions. *P. carotovorum* subsp. *carotovorum* probably possesses the broadest host range among soft-rot erwinias that includes potato, Brussels sprout, carrot, celery, cucumber, capsicum, turnip, chicory, konjac, and many other crops subject to postharvest rot caused by this pathogen [78,79]. The pathogen is found on plant surfaces and in soil. Inside the plant, the bacteria inhabit the vascular tissues and intercellular spaces of parenchymatous tissues until suitable environmental conditions for disease progress arise, including adequate moisture, oxygen levels, and temperature [78]. The pathogenesis of *P. carotovorum* subsp. *carotovorum* and other soft-rot erwinias relies on the production of multiple plant cell wall-degrading enzymes. These exoenzymes, such as pectinases, proteases, and cellulases, are actively secreted by the bacteria to break down plant cell walls and release nutrients that support bacterial growth [80].

In general, the activity of meadowsweet γ-core peptides against bacteria was lower than that against yeasts (Table 3). *P. carotovorum* was insensitive to the γ-core peptides at the tested concentrations. *C. michiganensis* was most effectively inhibited by γ_48-67_FuSN6, with IC_50_ = 34.7 μM. Conversely, the Gram-negative bacterium *P. savastanoi* was most efficiently inhibited by the defensin-derived γ-core peptides FuD0, with IC_50_ = 55.2 μM, followed by γ_59-74_FuDEFL1-1 (IC_50_ = 88.3 μM). Although γ_59-74_FuDEFL1-1 has a higher positive charge, it is less hydrophobic than FuD0, suggesting that hydrophobicity plays a more significant role in the activity against *P. savastanoi* than the charge.

The γ-core peptides were assayed against four *Fusarium* species: *F. oxysporum*, *F. solani*, *F culmorum*, and *F. verticillioides*.

The *Fusarium* genus is a highly diverse group of filamentous ascomycete fungi, comprising approximately 300 phylogenetically distinct species widely distributed in soil environments [81]. Many strains are non-pathogenic, while others are plant and animal pathogens. Pathogenic *Fusarium* fungi primarily affect various plants, leading to significant agricultural losses. In addition, *Fusarium* spp. produce harmful mycotoxins like trichothecenes, fumonisins, zearalenones, etc., posing health risks to animals and humans. These toxic compounds are transferred to food products and cause different diseases in humans, such as organ failure, cancer, and hormonal abnormalities. Among pathogenic *Fusarium* species, *F. oxysporum* is notably the most economically important, causing Fusarium wilt diseases in a broad range of crops such as tomatoes, bananas, sweet potatoes, onions, and legumes, as well as ornamental plants [82,83]. Many *F. oxysporum* isolates are host-specific. The disease symptoms in plants include the browning of vascular tissues, necrosis, defoliation, and eventual plant death, highlighting the substantial impact of this pathogen on agricultural productivity [82]. *F. solani* as a plant pathogen affects a diverse range of hosts, including vegetables, woody species, and ornamental plants [84]. It induces root rot, stem dieback, and soft rot diseases, often leading to significant crop losses. *F. solani* produces multiple macroconidia and microconidia, contributing to its high adaptability and pathogenic potential. This species is able to survive in soil for extended periods and is resistant to environmental stresses, making management challenging. It is also capable of producing mycotoxins that can impact both plant health and human and animal health. *F. culmorum* is a ubiquitous soil-borne fungal pathogen primarily affecting small-grain cereals, such as wheat and barley. *F. culmorum* is a causal agent of foot and root rot (Fusariun crown rot) and Fusarium head blight [85]. The diseases lead to reduced yield and grain quality, due to contamination with harmful mycotoxins, such as deoxynivalenol and nivalenol, which pose health risks to humans and animals. *F. culmorum* is found in temperate regions. Its pathogenicity is influenced by environmental conditions, such as humidity and temperature. Management strategies include crop rotation, resistant varieties, and fungicide application. *F. verticillioides* is a globally distributed plant pathogen, especially in humid tropical and subtropical regions. The fungus primarily affects crops such as maize, sorghum, rice, millet, and others [86]. It causes various diseases including stalk and cob rot in maize, foot rot in rice, crown rot in asparagus, and top rot in sugarcane. Its infection not only reduces crop yields and grain quality but also leads to contamination with fumonisins, toxic compounds posing food safety risks for humans and animals.

*Fusarium* species are the second most common non-*Aspergillus* molds causing human infections in the US and Europe, with the *F. solani* complex accounting for about 50% of cases, followed by *F. oxysporum* (~20%) and *F. verticillioides* and *F. moniliforme* (~10% each) [87,88]. Infections typically enter through the airways or skin, especially in immunocompromised individuals with neutropenia or T-cell deficiencies, leading to severe conditions like pneumonia, sinusitis, and fungemia with high mortality rates (up to 94% in 12 weeks). In immunocompetent hosts, keratitis and onychomycosis are more common [89,90]. The outbreaks of fusarial keratitis among contact lens wearers and peritonitis in dialysis patients were described, highlighting the pathogen’s clinical significance [91]. *Fusarium* species are capable of forming biofilms, making these pathogens resistant to current antifungal treatments and difficult to cure. Instances of *Fusarium* infections have increased in recent decades. In 2022—2023, a severe outbreak of *F. solani* meningitis among immunocompetent individuals was recorded in Mexico and the USA [92].

Studies of the antimicrobial activity of the γ-core peptides against *Fusarium* fungi showed that both the snakin- and defensin-derived peptides displayed potent inhibitory activity, except for γ_88-104_FuSN3 with the lowest net charge among the peptides (Table 4). There was a considerable variation in the activity of different peptides against diverse *Fusarium* species. *F. oxysporum* and *F. culmorum* were the most responsive species. γ_48-67_FuSN6 and γ_59-74_FuDEFL1-1 exhibited the highest potency against three *Fusarium* species—*F. culmorum*, *F. oxysporum*, and *F. verticillioides*. FuD0 was the most efficient inhibitor of *F. solani* and *F. oxysporum* as well (IC_50_ = 17.7 and 11.3 μM, respectively). γ_59-74_FuDEFL1-1 and γ_48-67_FuSN6 were the most active peptides against *F. verticillioides*.

The γ-core peptides were also assayed against the important pathogen of cereals *B. sorokiniana* and necrotrophic fungal pathogen *B. cinerea*.

*B. sorokiniana* primarily affects small grain cereals like wheat and barley, causing diseases such as common root rot, leaf spot, seedling blight, head blight, and black point, with rye being less susceptible and oats rarely infected [93]. The fungus induces dark brown necrotic lesions on roots, crowns, and lower leaf sheaths. In severe cases, plants dry out without seed production, especially under conditions of high temperature and humidity prevalent in South Asia, leading to significant yield losses. In South Asia, the disease complex involving spot blotch and tan spot (caused by *Pyrenophora tritici*–*repentis*) results in notable crop damage, with losses reaching 16–23% across India, Nepal, and Bangladesh. Spot blotch, caused by *B. sorokiniana*, is widespread in wheat-growing regions and can lead to substantial yield losses of 15–25% in warm areas, while seed infection can also trigger black point disease, further resulting in root rot and seedling blight, thereby significantly impacting crop health and productivity [94].

*B. cinerea*, or grey mold, is a highly adaptable necrotrophic pathogen within Ascomycota that infects over 600 plant genera, including eudicots, monocots, basal angiosperms, gymnosperms, pteridophytes, and bryophytes, making it one of the most economically damaging phytopathogens globally [82]. Its success as a pathogen stems from prolific reproduction, survival strategies, and diverse infection mechanisms, leading to widespread plant tissue necrosis and rot [95]. Disease management is challenging due to the pathogen’s rapid development of fungicide resistance, with chemical control being the most common approach, underscoring the need for alternative strategies.

The most efficient inhibitors of *B. sorokiniana* were γ_59-74_FuDEFL1-1, γ_48-67_FuSN6, and FuD0. They suppressed growth of the pathogen with IC_50_ values of 45.0, 46.2, and 51.7 μM, respectively. *B. cinerea* was found to be quite resistant to the peptides. γ_88-104_FuSN3 appeared to be the most efficient inhibitor of the fungus. The peptide suppressed the pathogen’s growth, with IC_50_ = 61.7 μM.

Thus, the antimicrobial assays allowed us to identify the most active γ-core peptides against particular human or plant pathogens. To shed light on the mode of action of the meadowsweet γ-core peptides, staining with propidium iodide was performed. The following pathogens were used: *C. albicans*, *C. tropicalis*, *C. neoformans*, and *C. michiganensis* (Figure 3). All of the synthetic peptides triggered membrane permeabilization of the yeast cells, suggesting that pathogen suppression involves disturbances in membrane properties. The results were invalid with *C. michiganensis* since the staining of the bacterial cells was poor even after electroporation or treatment with 50 μM AgNO_3_.

We further studied possible synergism between the synthetic peptides. We chose two peptide pairs—a pair of defensin-derived peptides (γ_59-74_FuDEFL1-1/γ_61-76_FuDEFL1-7) and a pair of defensin- and snakin-derived peptides (FuD0/γ_54-69_FuSN5). Synergism was measured against the fungus *F. oxysporum* and the bacterium *C. michiganensis* (Table 5 and Table 6). The study revealed that the peptide combinations γ_59-74_FuDEFL1-1/γ_61-76_FuDEFL1-7 and FuD0/γ_54-69_FuSN5 exhibited synergistic antifungal activity against *F. oxysporum* at certain concentrations, enhancing pathogen suppression beyond additive effects, whereas against *C. michiganensis*, these combinations only demonstrated additive effects, indicating that the mode of interaction between the peptides and pathogens varies depending on the microbial target. The data obtained on the synergistic action of the γ-core peptides derived from the AMPs complement our previous findings regarding the synergy between the oligopeptides derived from the hevein-like peptide WAMP and the fungicide tebuconazole [96,97]. The potential mechanisms of synergy may include complementary membrane targeting, pore formation cooperativity, etc. However, the exact mechanisms of synergistic effects between peptides require further studies.

In summary, our study highlights *F. ulmaria* as a rich source not only of secondary metabolites but of biologically active polypeptide compounds with strong antimicrobial properties as well. The synthetic γ-core peptides—shortened, functionally essential fragments of the meadowsweet AMPs that retain biological activity and can act synergistically with each other and conventional antimicrobials, present promising templates for developing new antimicrobial agents against diverse plant and human opportunistic pathogens that are safe for the environment and human health, and the use of which will reduce the amounts of toxic pesticides used.

## 4. Materials and Methods

### 4.1. Chemical Synthesis of Short Peptides Derived from AMP-like Peptides

Seven peptide fragments of meadowsweet and tomato AMP-like peptides belonging to the DEFL and snakin families were generated by solid-phase synthesis using the Fmoc method (Elabscience Biotechnology Inc., Wuhan, China). The synthetic peptides were purified by RP-HPLC. Their identity to the required sequences was confirmed by matrix-assisted laser desorption/ionization time-of-flight (MALDI-TOF) mass spectrometric analysis using the Ultraflex MALDI-TOF mass spectrometer (Bruker Daltonics, Bremen, Germany) in a linear or reflector positive-ion mode using α-cyano-4-hydroxycinnamic acid as a matrix.

The following characteristics of the synthesized peptides were predicted using the ExPASy ProtParam tool [98]: molecular weight, net charge at pH 7, pI, GRAVY index, and aliphatic index. Boman index, hydrophobicity, and ratio of hydrophobic residues were computed using APD3 [99]. Prediction of antimicrobial properties was carried out with CAMPR4 [100].

### 4.2. Three-Dimensional Structure Modeling

The 3D structure of the synthetic peptides was de novo modeled using the PEP-FOLD3 program [47]. The best representative models were considered those which had the lowest sOPEP values provided by PEP-FOLD3.

### 4.3. Antimicrobial Assays

#### 4.3.1. Antimicrobial Activity Assays Against Bacteria and Yeasts

The antimicrobial activity of synthetic peptides was assayed against the yeasts *Cryptococcus neoformans* VKM Y-2755, *Candida albicans* VKM Y-2994, and *C. tropicalis* VKM Y-2771, as well as against the bacteria *Pectobacterium carotovorum* subsp. *carotovorum* VKM B-1247, *Pseudomonas savastanoi* pv. *savastanoi* VKM B-1546, and *Clavibacter michiganensis* subsp. *michiganensis* VKM Ac-1403 (All-Russian Collection of Microorganisms (VKM), Pushchino, Russia). Yeasts were grown on the YPD-P medium that contained (g/L): glucose—10, peptone—5, and yeast extract—4. Bacterial cultures were cultivated on the medium containing (g/L): yeast extract—1, soya extract—30, aminopeptides (a solution of low-molecular-weight peptides and all essential amino acids obtained from the blood of cattle by enzymatic hydrolysis)—60, tryptone—5, and pH 7.2. The microbial cultures were grown on a shaker at 30 °C for 24 h. The optical density of the cultures was measured on a UNICO 1201 (Unico, Princeton, NJ, USA) spectrophotometer at 594 nm. Pathogen cultures were diluted 1:20 with water before analysis. With *P. carotovorum* and *C. michiganensis*, a modified YPD-P medium was employed. The medium contained (g/L): glucose—5, peptone—10, and yeast extract—5; and for the experiments with *P. savastanoi*, the medium did not contain glucose, and the concentrations of peptone and yeast extract were 20 g/L and 10 g/L, respectively. The antimicrobial activity of peptides against yeasts and bacteria was assayed in immunoassay microtiter plates. A total of 10 µL of the tested peptide solution (final concentrations of 0.5–300 µM) in water, 10 µL of the microbial suspension, and 80 µL of the medium were mixed in a well of the microtiter plate and incubated at 30 °C for 24 h. After that, the absorbance of the suspension at 594 nm was measured on a Efos 9305 spectrophotometer (Sapphire, Moscow, Russia). For each pathogen, antimicrobial assays were carried out in triplicate per treatment. The peptide concentration necessary for 50% inhibition of the pathogen growth (IC_50_) was estimated from dose–response curves. The IC_50_ values are presented as mean ± standard deviations.

#### 4.3.2. Antifungal Activity Assays

The inhibitory activity of peptides on spore germination and growth of fungi was tested against nine fungal strains from the VKM: *Fusarium culmorum* VKM F-2303, *F. oxysporum* VKM F-137, *F. solani* VKM F-142, *F. verticillioides* VKM F-670, *Aspergillus unguis* VKM F-1754, *Bipolaris sorokiniana* VKM F-4006, *Botrytis cinerea* VKM F-4549, *Rhizoctonia solani* VKM F-895, and *Penicillium gladioli* VKM F-2088. The fungi were cultivated on potato dextrose agar at 25 °C for 7–8 days, while *B. sorokiniana* and *B. cinerea* were grown for 10–12 days. Spores were obtained by washing from the surface of the mycelia with sterile distilled water and filtered through a sterile filter. The filtrate was centrifuged at 6000 rpm for 15 min. The precipitate was washed three times with sterile distilled water and centrifuged at 10,000 rpm for 2 min. A total of 1 mL of sterile aqueous 20% glycerol solution was added to the spores, and spores were stored at −20 °C.

The antifungal effect of peptides was determined in microtiter plates by measuring the absorbance of the spore suspension in the presence of the peptide relative to the control. In the wells of a plate, 90 µL of spore suspension in half-strength potato dextrose broth at a concentration of 2000–3000 spores in 100 µL was mixed with 10 µL of aqueous solutions of peptides to obtain final concentrations of 5–300 µM. Each experiment was carried out in triplicate. The concentration of spores in the suspension was determined in a Goryaev chamber. The absorbance was recorded at 595 nm on a FilterMax F5 Multi-Mode Microplate Reader (Molecular Devices, San Jose, CA, USA) after 48 h of incubation. The antimicrobial activity was expressed in IC_50_ values.

#### 4.3.3. Synergistic Interaction Assays

The antimicrobial activity of pairs of synthetic peptides was studied in co-applications against the fungus *F. oxysporum* and the bacterium *C. michiganensis*. Cultivation of pathogens and the study of the effect of peptides on their growth in microtiter plates were carried out as described above, with the following modification: A total of 5 µL of aqueous solutions of each peptide in different combinations of concentrations was added to the wells. For the experiments with *F. oxysporum*, the final peptide concentrations of 0, 5, 10, 25, 50, 75, 100, and 150 µM were used; for the experiments with *C. michiganensis*, the peptide concentrations were 0, 10, 25, 50, 75, and 100 μM. Experiments were performed in triplicate. The percentage of pathogen growth inhibition was calculated relative to the control (growth without peptide addition).

To reveal synergy of pairs of the γ-core peptides, the Limpel’s criterion Ee < Er [101] was determined by Formula (1):

Ee, % = (X + Y) − XY / 100 < Er, % (at *p* ≤ 0.05), (1)

where Ee denotes the level of an expected additive effect from application of both tested compounds; X and Y show inhibition of spore germination in % by each peptide used individually; and Er represents the percentage of inhibition obtained experimentally by simultaneous application of X and Y.

### 4.4. Statistical Analysis

Mean values, standard deviations (SD), and the significance of differences (*p* ≤ 0.05) of the means between treatments and controls (*t*-test for independent variables) were determined with the Microsoft Excel program (Microsoft, Redmond, WA, USA).

### 4.5. Staining of Pathogen Cells with Propidium Iodide

Three yeast and one bacterium pathogens were chosen for analysis. *C. albicans*, *C. tropicalis*, and *C. neoformans* were grown on the YPD-P medium at 28 °C for 24 h, 24 h, and 48 h, respectively. *C. michiganensis* was grown on a modified YPD-P medium, which contained (g/L) glucose—5, peptone—10, and yeast extract—5, at 28 °C for 48 h. Pathogen cultures were diluted 1:20 with water and incubated with the peptide at a concentration of 100 or 200 µM at 30 °C for 1.5 h. The cells were stained with propidium iodide (Sigma-Aldrich, St. Louis, MO, USA) by direct addition of the dye to the incubation medium (the final concentration of 3 µg/mL) and after brief (1–2 min) incubation at room temperature measured on a NovoCyte Flow cytometer (Agilent, Santa Clara, CA, USA) using 488 nm for excitation and 572/28 nm for emission (a total of 100,000 cells were counted for each sample). The samples without peptides were used as a control. All assays were repeated three times, and the mean results are presented.

## 5. Conclusions

In the present work, we examined the antimicrobial properties against a broad spectrum of plant and human pathogens of several γ-core peptides derived from *Filipendula ulmaria* AMPs. We discovered the peptides with potent activity against particular pathogens. Thus, our study highlights *F. ulmaria* as a rich source not only of secondary metabolites but of biologically active polypeptide compounds with strong antimicrobial properties as well. We also revealed synergism of action between meadowsweet peptides and showed that membrane permeabilization contributes to the peptides’ mode of action. These short peptides are derived from natural AMPs, the evolutionary ancient players in plant immunity discovered in all forms of life. They exhibit potent antimicrobial activity, in some cases exceeding that of the intact peptide. Being shortened versions of the parent AMPs, they surpass them in bioavailability and offer cost-effective production by solid-phase synthesis, which opens new avenues for commercial use of these peptide products as novel antimicrobials in agricultural sector and medicine.

## Figures and Tables

**Table 1:** Sequences of synthetic peptides.

Peptide Name	Amino Acid Sequence
γ_59-74_FuDEFL1-1	GKCSHILRRCRCSKSC
γ_61-76_FuDEFL1-7	GKCSHILRRCQCLKSC
FuD0	GKCSHILRRCFCTTHC
γ_88-104_FuSN3	GSCYTDMTTHGNRLKCP
γ_48-67_FuSN6	GACIARCRLSSRPNLCKR
γ_54-69_FuSN5	GRCKLSSRPKLCKR
γ_89-106_SlSN9 *	GLCKYRCSLHSRPNVCFR

* The peptide is described in [45].

**Table 2:** Physicochemical properties of meadowsweet AMP-based synthetic peptides.

Peptide	Length, aa	Molecular Weight, Da	Net Charge at pH 7	pI	Aliphatic Index	Boman Index	Ratio of Hydrophobic Residues, %	GRAVY Index	AMP Prediction
γ_88-104_FuSN3	17	1884.13	+1	8.05	22.94	2.35	24	−0.853	Non-AMP
γ_48-67_FuSN6	18	2004.42	+5	10.74	76.11	3.08	44	−0.322	AMP
γ_54-69_FuSN5	14	1632.02	+6	11.02	55.71	3.91	29	−1.157	AMP
γ_59-74_FuDEFL1-1	16	1837.23	+5	9.70	48.75	3.42	38	−0.562	AMP
γ_61-76_FuDEFL1-7	16	1835.25	+4	9.31	73.12	2.31	44	−0.212	AMP
FuD0	16	1865.24	+3	8.98	48.75	2.14	44	−0.050	AMP

**Table 3:** Antimicrobial activity of the γ-core motif peptides against yeasts and bacteria *.

Pathogen	IC_50_, μM						
	γ_59-74_FuDEFL1-1	γ_61-76_FuDEFL1-7	FuD0	γ_88-104_FuSN3	γ_48-67_FuSN6	γ_54-69_FuSN5	γ_89-106_SlSN9
*C. neoformans*	43.5 ± 11.6	2.7 ± 0.2	6.1 ± 0.0	152.2 ± 5.6	5.5 ± 0.2	1.4 ± 0.7	6.3 ± 3.8
*C. albicans*	13.8 ± 1.4	100.6 ± 23.4	41.8 ± 3.6	–	–	10.4 ± 4.0	214.3 ± 47.1
*C. tropicalis*	7.9 ± 0.7	3.6 ± 1.8	5.1 ± 1.3	185.0 ± 2.5	1.7 ± 0.6	2.5 ± 0.2	6.1 ± 0.1
*C. michiganensis*	198.5 ± 1.4	198.1 ± 2.5	134.1 ± 3.1	–	34.7 ± 0.5	126.9 ± 0.3	41.9 ± 0.5
*P. savastanoi*	88.3 ± 0.4	127.2 ± 0.6	55.2 ± 1.7	–	97.2 ± 0.2	97.9 ± 0.5	127.6 ± 0.9
*P. carotovorum*	–	–	–	–	–	–	–

* Mean values ± SD are presented; “–” indicates no activity at peptide concentrations below 300 μM.

**Table 4:** Antifungal activity of the γ-core motif peptides *.

Pathogen	IC_50_, μM						
	γ_59-74_FuDEFL1-1	γ_61-76_FuDEFL1-7	FuD0	γ_88-104_FuSN3	γ_48-67_FuSN6	γ_54-69_FuSN5	γ_89-106_SlSN9
*F. culmorum*	19.4 ± 3.7	47.0 ± 8.5	36.2 ± 0.8	–	19.3 ± 1.2	25.3 ± 12.2	23.1 ± 4.5
*F. oxysporum*	22.2 ± 0.7	38.3 ± 0.5	11.3 ± 0.7	–	15.8 ± 0.6	116.2 ± 18.5	34.1 ± 1.0
*F. solani*	49.0 ± 2.3	64.5 ± 2.7	17.7 ± 1.5	–	51.4 ± 0.9	111.8 ± 32.5	50.5 ± 1.4
*F. verticillioides*	36.1 ± 6.0	56.0 ± 4.2	43.1 ± 1.6	174.6 ± 8.1	38.0 ± 4.6	72.3 ± 12.8	32.3 ± 6.5
*A. unguis*	28.3 ± 2.8	37.0 ± 1.0	18.0 ± 2.0	2.2 ± 0.1	2.1 ± 0.0	27.2 ± 2.8	3.3 ± 0.2
*P. gladioli*	67.3 ± 3.3	113.4 ± 25.2	94.1 ± 8.3	69.7 ± 0.1	93.8 ± 4.2	115.7 ± 31.1	93.6 ± 5.2
*B. sorokiniana*	45.0 ± 10.2	63.0 ± 7.8	51.7 ± 4.6	103.0 ± 8.1	46.2 ± 3.2	–	55.1 ± 7.0
*B. cinerea*	93.2 ± 1.2	n/d	110.9 ± 1.7	61.7 ± 0.5	154.2 ± 4.4	–	117.8 ± 12.0
*R. solani*	n/d	n/d	102.5 ± 4.2	123.9 ± 6.9	n/d	–	129.5 ± 2.9

* Mean values ± SD are presented; “–” indicates no activity at peptide concentrations below 300 μM; n/d means not determined.

**Table 5:** Observed inhibitory effect (Er, %) on *F. oxysporum* growth of the γ_59-74_FuDEFL1-1/γ_61-76_FuDEFL1-7 pair compared to the calculated additive inhibitory effect (Ee, %). Er values of peptide concentration combinations for which Ee < Er are shown in bold. Er values of peptide concentration combinations for which Ee < Er is fulfilled at *p* ≤ 0.05 are marked with an asterisk.

Concentration of γ59-74FuDEFL1-1, μM	Concentration of γ61-76FuDEFL1-7, μM												
	0	5		10		25		50		75		100	
	Er	Er	Ee	Er	Ee	Er	Ee	Er	Ee	Er	Ee	Er	Ee
0		1.5		5.0		25.0		67.4		79.5		61.6	
5	5.0	**12.3**	6.4	**32.0**	9.7	86.1 *	28.7	**80.8**	69.0	**84.3**	80.5	**82.0**	63.5
10	3.8	**27.3**	5.2	**37.1**	8.6	80.3 *	27.9	**83.6**	68.6	80.0	80.3	**81.3**	63.1
25	64.7	82.8 *	65.3	**79.0**	66.5	**84.4**	73.6	82.3	88.5	82.1	92.8	78.6	86.5
50	74.5	**82.6**	74.9	**84.1**	75.8	83.3	80.9	81.6	91.7	80.3	94.8	81.5	90.2
75	79.1	**82.1**	79.4	**83.3**	80.2	83.8	84.4	80.5	93.2	78.3	95.7	76.2	92.0
100	77.5	**81.0**	77.8	**81.3**	78.6	79.3	83.1	78.3	92.7	76.2	95.4	74.3	91.4

**Table 6:** Observed inhibitory effect (Er, %) on *F. oxysporum* growth of the FuD0/γ_54-69_FuSN5 pair compared to the calculated additive inhibitory effect (Ee, %). Er values of peptide concentration combinations for which Ee < Er are shown in bold. Er values of peptide concentration combinations for which Ee < Er is fulfilled at *p* ≤ 0.05 are marked with an asterisk.

Concentration of FuD0, μM	Concentration of γ54-69FuSN5, μM														
	0	5		10		25		50		75		100		150	
	Er	Er	Ee	Er	Ee	Er	Ee	Er	Ee	Er	Ee	Er	Ee	Er	Ee
0		11.7		10.7		17.5		13.7		6.9		43.5		54.7	
5	9.2	8.1	19.8	9.1	18.9	17.8	25.1	**46.1**	21.6	49.6 *	15.5	**67.4**	48.7	**79.1**	58.9
10	18.6	1.0	28.1	5.4	27.3	2.8	32.8	**30.6**	29.7	**43.3**	24.2	53.9	54.0	**69.9**	63.1
25	64.9	**69.0**	69.0	**73.8**	68.7	**75.1**	71.0	69.2	69.7	**70.3**	67.3	70.8	80.2	83.5	84.1
50	79.4	77.6	81.8	78.9	81.6	**84.8**	83.0	81.9	82.2	**81.4**	80.8	78.1	88.4	83.5	90.7
75	81.9	79.4	84.0	80.4	83.8	84.3	85.0	81.4	84.4	81.2	83.1	81.2	89.8	80.9	91.8
100	84.5	81.1	86.3	84.0	86.2	81.7	87.2	81.2	86.6	80.9	85.6	81.1	91.2	81.5	93.0

**Table 7:** Observed inhibitory effect (Er, %) on *C. michiganensis* of the γ_59-74_FuDEFL1-1/γ_61-76_FuDEFL1-7 pair compared to the calculated additive inhibitory effect (Ee, %).

Concentration of γ59-74FuDEFL1-1, μM	Concentration of γ61-76FuDEFL1-7, μM										
	0	10		25		50		75		100	
	Er	Er	Ee	Er	Ee	Er	Ee	Er	Ee	Er	Ee
0		3.4		7.0		16.4		21.6		24.2	
10	2.4	2.7	5.7	3.9	9.2	7.5	18.5	9.9	23.5	14.5	26.0
25	5.6	8.0	8.8	10.3	12.2	11.2	21.1	11.5	26.0	16.1	28.4
50	14.9	11.4	17.8	13.6	20.8	13.7	28.9	14.9	33.3	23.4	35.5
75	20.5	13.9	23.2	17.7	26.1	20.9	33.6	21.8	37.7	32.1	39.8
100	23.8	14.8	26.3	19.7	29.1	23.8	36.3	25.5	40.2	36.6	42.2

**Table 8:** Observed inhibitory effect (Er, %) on *C. michiganensis* of the FuD0/γ_54-69_FuSN5 pair compared to the calculated additive inhibitory effect (Ee, %).

Concentration of FuD0, μM	Concentration of γ54-69FuSN5, μM										
	0	10		25		50		75		100	
	Er	Er	Ee	Er	Ee	Er	Ee	Er	Ee	Er	Ee
0		16.9		26.3		30.6		34.7		43.2	
10	4.7	20.1	20.8	28.2	29.7	30.4	33.8	31.4	37.7	32.2	45.8
25	16.8	25.8	30.8	34.8	38.7	35.0	42.2	35.8	45.6	43.5	52.7
50	24.0	32.1	36.8	36.3	44.0	37.0	47.2	43.8	50.3	45.4	56.8
75	28.3	32.9	40.4	37.4	47.2	38.1	50.2	45.0	53.2	46.4	59.3
100	38.1	43.3	48.5	44.7	54.4	44.9	57.0	49.7	59.6	50.8	64.8

## Data Availability

The original contributions presented in this study are included in the article. Further inquiries can be directed to the corresponding author.

## Abbreviations

The following abbreviations are used in this manuscript:

AMP	Antimicrobial peptide
DEFL	Defensin-like peptide
pI	Isoelectric point
GRAVY	Grand average of hydropathy
IC_50_	Peptide concentration required for 50% inhibition of the pathogen growth
SD	Standard deviation
PI	Propidium iodide
